# Ascorbic acid as an adjunctive therapy in critically ill patients with COVID-19: a propensity score matched study

**DOI:** 10.1038/s41598-021-96703-y

**Published:** 2021-09-03

**Authors:** Khalid Al Sulaiman, Ohoud Aljuhani, Khalid Bin Saleh, Hisham A. Badreldin, Abdullah Al Harthi, Mohammed Alenazi, Aisha Alharbi, Rahmah Algarni, Shmeylan Al Harbi, Abdullah M. Alhammad, Ramesh Vishwakarma, Sarah Aldekhyl

**Affiliations:** 1 Pharmaceutical Care Department, King Abdulaziz Medical City, Riyadh, Saudi Arabia; 2 Department of Pharmacy Practice, Faculty of Pharmacy, King Abdulaziz University, Jeddah, Saudi Arabia; 3 Pharmaceutical Care Department, King Abdulaziz University Hospital, Jeddah, Saudi Arabia; 4 College of Medicine, King Saud Bin Abdulaziz University for Health Sciences, King Abdullah International Medical Research Center, Riyadh, Saudi Arabia; 5 Intensive Care Department, King Abdulaziz Medical City, Riyadh, Saudi Arabia; 6 College of Pharmacy, King Saud Bin Abdulaziz University for Health Sciences, Riyadh, Saudi Arabia; 7 Biostatistics and Bioinformatics Department, King Abdullah International Medical Research Center, Riyadh, Saudi Arabia; 8 Department of Clinical Pharmacy, College of Pharmacy, King Saud University, Riyadh, Saudi Arabia; 9 King Abdulaziz Medical City (KAMC)-Ministry of National Guard Health Affairs (MNGHA), King Abdullah International Medical Research Center/King Saud bin Abdulaziz University for Health Sciences, PO Box 22490, Riyadh, 11426 Saudi Arabia

**Keywords:** Outcomes research, Infectious diseases

## Abstract

Ascorbic acid represents an appealing option for clinicians to utilize in the context of the global COVID-19 pandemic due to its proposed clinical efficacy, relative safety, and low cost. The aim of this study was to evaluate the efficacy and safety of using ascorbic acid in supplemental doses as adjunctive therapy for patients critically ill with COVID-19. This was a two-center, non-interventional, retrospective cohort study. All critically ill adult patients admitted to ICU with a confirmed COVID-19 diagnosis between March 1st and December 31st, 2020, were included in the final analysis. The study was conducted at two large governmental tertiary hospitals in Saudi Arabia. The purpose was to investigate the clinical outcomes of low-dose ascorbic acid as adjunctive therapy in COVID-19 after propensity score matching using baseline severity scores, systematic use of corticosteroids, and study centers. A number of 739 patients were included in this study, among whom 296 patients were included after propensity score matching. There was no association between the administration of ascorbic acid and in-hospital mortality or the 30-day mortality [OR (95% CI) 0.77 (0.47, 1.23), p value = 0.27 and OR (95% CI) 0.73 (0.43, 1.20), p value = 0.21, respectively]. Using ascorbic acid was associated with a lower incidence of thrombosis compared with the non-ascorbic-acid group [6.1% vs. 13% respectively; OR (95% CI) 0.42 (0.184, 0.937), p value = 0.03]. Low dose of ascorbic acid as an adjunctive therapy in COVID-19 critically ill patients was not associated with mortality benefits, but it was associated with a lower incidence of thrombosis. Further studies are required to confirm these findings.

## Introduction

The severe acute respiratory syndrome caused by the novel coronavirus 2 (SARS-CoV-2) represents one of the most recent serious healthcare challenges of humanity. To date, most of the available investigated treatments are supportive measures with few proposed preventive measures^1^. However, there are some agents proposed to have a role in the treatment and prevention of coronavirus disease of 2019 (COVID-19). Due to its known antioxidant effects and role in enhancing immune function, ascorbic acid (Vitamin C) was assumed to have a beneficial impact on COVID-19. This is mainly via supporting lymphocyte activity, stimulating interferon-α production, reducing inflammation, and improving endothelial function.^2–4^.

Ascorbic acid is a water-soluble vitamin that is believed to have clinical benefits for patients with severe illnesses. The antioxidant properties of ascorbic acid have been evaluated in severe oxidative stress statuses such as serious infection, sepsis, and acute respiratory distress syndrome (ARDS). COVID-19 infection can lead to serious oxidative stress leading to a state where patients might require more ascorbic acid. Several reports addressed ascorbic acid's potential effect in ameliorating inflammation and vascular injury in critically ill patients^5,6^. In light of its proposed efficacy, relative safety, and low cost, ascorbic acid represents an appealing agent for researchers and clinicians to utilize in the context of a global health pandemic.

Several studies have mixed results regarding the clinical use of ascorbic acid in non-COVID-19 critically ill patients. A pilot study compared intravenous (IV) ascorbic acid with a placebo arm in 24 critically ill patients with sepsis. This study showed that patients who received IV ascorbic acid had lower sequential organ failure assessment (SOFA) scores and lower levels of pro-inflammatory markers compared to the placebo group^7^. Another randomized controlled study conducted in 167 critically ill patients with sepsis-induced ARDS found no difference in SOFA scores and levels of inflammatory markers between the groups. However, the 28-day mortality was lower in the treatment group^8^.

Multiple studies have evaluated ascorbic acid in non-COVID-19 critically ill patients^9–12^. One meta-analysis that evaluated ascorbic acid use in intensive care unit (ICU) patients without COVID-19 found that high-dose IV ascorbic acid infusions (i.e., 200 mg/kg/day) shortened the ICU length of stay by 7.8%^11^. A recent report investigating using high-dose IV ascorbic acid to treat 50 moderate to severe COVID-19 patients showed an improvement in the oxygenation index^12^. Given the scarcity of published data to investigate the effect of ascorbic acid in critically ill patients with COVID-19, various dosing regimens, routes, and duration of treatment in non-COVID critically ill patients, we aimed to investigate the safety and efficacy of a low dose enteral ascorbic acid as adjunctive therapy in COVID-19 critically ill patients.

## Methods

### Study design

The study is a retrospective study of critically ill patients admitted to ICUs with a confirmed diagnosis of COVID-19 in two tertiary care centers in Saudi Arabia from March 1, 2020, to December 31, 2020. The diagnosis of COVID-19 was confirmed by reverse transcriptase polymerase chain reaction (RT-PCR) on nasopharyngeal and/or throat swabs. All the patients who met our inclusion criteria during the study period were included. Patients were divided into 2 groups based on ascorbic acid use as adjunctive therapy during ICU stay. All patients were followed until they were discharged from the hospital or died during the in-hospital stay, whichever occurred first.

### Eligibility criteria

Adults patients (≥ 18 years old) were enrolled in the study if they were admitted to the ICU with a confirmed diagnosis of COVID-19 using the PCR test. Patients were excluded if the ICU length of stay (LOS) was less than 24 h or labeled as "Do-Not-Resuscitate" status within 24 h of ICU admission.

### Setting

This study was conducted in two tertiary governmental hospitals; King Abdulaziz Medical City, Riyadh, and King Abdulaziz University Hospital, Jeddah. The primary site for this multicenter study was King Abdulaziz Medical City (Riyadh).

### Data collection

The following information was collected: demographic data (see additional file 1), comorbidities, vital signs, severity baseline scores (i.e., Acute Physiology and Chronic Health Evaluation II (APACHE II), Sequential Organ Failure Assessment (SOFA), and Nutrition Risk in Critically ill (NUTRIC)), Glasgow Coma Score (GCS), acute kidney injury (AKI), needs for mechanical ventilation (MV) and MV settings within 24 h of ICU admission. Additionally, laboratory tests such as renal profile, liver function tests (LFTs), coagulation profile (i.e., INR, aPTT, fibrinogen), and inflammatory markers (C-reactive protein (CRP), procalcitonin) within 24 h of ICU admission were collected. Lastly, the use of pharmacological venous thromboembolism (VTE) prophylaxis, corticosteroids and tocilizumab were recorded for the eligible patients and followed due to their potential benefits.

### Endpoints

The primary endpoint was estimating the in-hospital mortality in critically ill patients with COVID-19 who received a supplemental dose of ascorbic acid as adjunctive therapy versus those who did not receive ascorbic acid. The secondary endpoints were the following, 30-days mortality, ICU LOS, hospital LOS, MV duration. We also reported the following complications during ICU stay: AKI, liver injury, respiratory failure, and thrombosis/infraction.

### Definition (s)

- Acute kidney injury (AKI) was defined using Acute Kidney Injury Network (AKIN) definition^13^, which is a sudden decrease of renal function within 48 h, defined by an increase in absolute SCr of at least 26.5 μmol/L (0.3 mg/dL) or by a percentage increase in SCr ≥ 50% (1.5 × baseline value).
- Liver injury was defined as alanine aminotransferase (ALT) exceeding 3 times the upper limit of normal or double in patients with elevated baseline ALT during stay.
- Respiratory failure was defined as either hypoxemic respiratory failure (PaO_2_ < 60 mmHg with a normal or low arterial carbon dioxide tension (PaCO_2_) or hypercapnic respiratory failure (PaCO_2_ > 50 mmHg) that required mechanical ventilation.
- Thrombosis/infarction was defined using the International Classification of Diseases, Tenth Revision, Clinical Modification (ICD10-CM) code (i.e., myocardial infarction [MI], ischemic stroke, pulmonary embolism, deep vein thrombosis) during ICU stay^14^.

### Statistical analysis

Categorical variables were reported using numbers and percentages. Continuous variables were reported using mean with standard deviation (SD) or median with interquartile range (IQR) when appropriate.

We compared categorical variables using the chi-squared or Fisher's exact test. Continuous variables were compared numerically using the Student's t test (for the normally distributed variables) and other quantitative variables with the Mann–Whitney U test (for the non-normally distributed variables). The normality assumptions were assessed for all numerical variables using a statistical test (i.e., Shapiro–Wilk test) and using graphical representation (i.e., histograms and Q–Q plots).

Baseline characteristics, baseline severity, and endpoint variables were compared between the two treatment groups. Multivariate logistic regression and generalized linear regression were used to find out the relationship between ascorbic acid use and different outcomes considered in this study. We assessed model fit using the Hosmer–Lemeshow goodness-of-fit test. The odds ratios (OR) and estimates with the 95% confidence intervals (CI) were reported for the associations.

Propensity score matching Procedure (Proc PS match) (SAS, Cary, NC) was used to match patients who received ascorbic acid to patients who did not, based on patients' baseline severity scores (i.e., APACHE II, SOFA score, NUTRIC scores), systematic use of corticosteroids, and study centers. A greedy nearest neighbor matching method was used in which one non-ascorbic acid (control) was matched with each patient in the ascorbic acid (active) group, which eventually produced the smallest within-pair difference among all available pairs with treated patients. Patients were matched only if the difference in the logits of the propensity scores for pairs of patients from the two groups was less than or equal to 0.5 times the pooled estimate of the standard deviation. ﻿Kaplan–Meier (KM) curves for the time to death were constructed censoring by hospital discharge or at 90 days, whichever occurred first. The log-rank test was used to compare the median survival time between the two groups. No imputation was made for missing data as the cohort of patients in our study was not derived from random selection. We considered a p value of < 0.05 statistically significant and used SAS version 9.4 for all statistical analyses.

## Results

A total of 739 patients met the inclusion criteria. Of those included, 158 (21.3%) patients received ascorbic acid while 581 (78.7%) patients did not. A total of 296 patients were included after propensity score matching based on the selected criteria^15^.

All included patients in the ascorbic acid group received a low-dose dose of ascorbic acid enterally (1000 mg once daily) with a median duration of administration of 11 days (IQR 7–18). Ascorbic acid was initiated within 24 h of ICU admission in (61.9%) of the patients.

### Demographic and clinical characteristics

The majority of the included patients in both arms were male (72%) with a mean age of 60.65 (SD ± 14.81). Before propensity score matching, the predominant underlying comorbidities were diabetes mellitus (59%) followed by hypertension (56%) and dyslipidemia (29%). Most of the comorbidities were similar between the two groups (Additional file 1).

Patients who didn't receive ascorbic acid as adjunctive therapy had higher baseline severity scores (i.e., APACHE II, SOFA, and NUTRIC scores), AKI, required MV within 24 h of ICU admission, and had higher baseline laboratory tests. Conversely, patients who received ascorbic acid as adjunctive therapy had significantly higher systematic corticosteroid use during ICU, estimated glomerular filtration rate (eGFR), and pH. Following the propensity score matching, most of these baseline and demographic characteristics were shown to be similar between the two groups (Additional file 1).

### Mortality and length of stay

**Figure 1:**
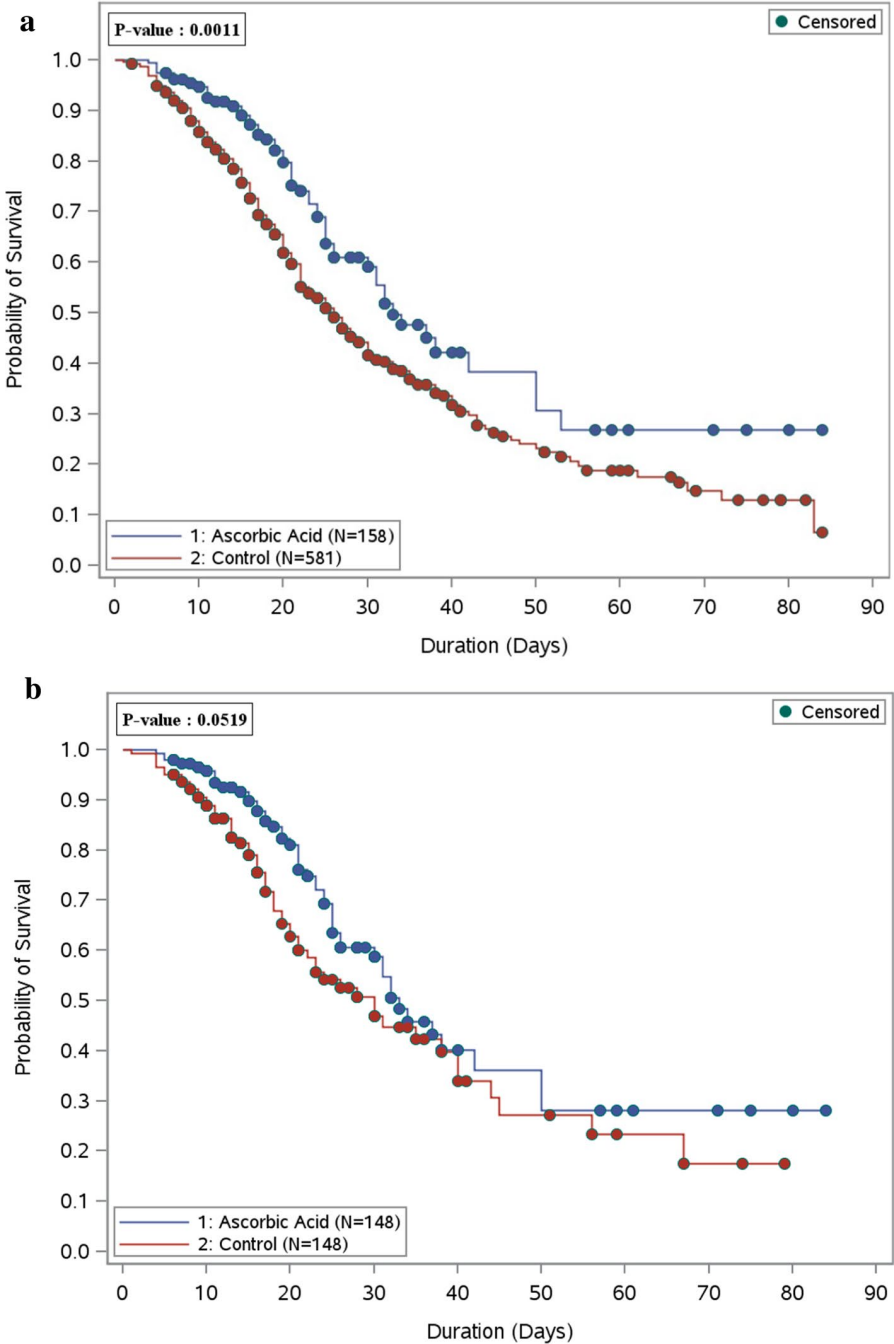
(**a**) Overall survival plot during the hospital stay comparing patients who received ascorbic acid (157 patients) as adjunctive therapy versus the control group (581 patients)—before PS matching. (**b**) Overall survival plot during the hospital stay comparing patients who received ascorbic acid (148 patients) as adjunctive therapy versus the control group (148 patients)—after PS matching.

During the hospital stay, the analysis for all eligible patients who received ascorbic acid showed significantly lower in-hospital mortality rates in comparison to the non-ascorbic acid group (33.6% vs. 49.3% respectively, p = 0.0006). However, after propensity matching, the difference between the two groups became statistically insignificant (32.4% in the ascorbic acid group vs. 41.6% in the non-ascorbic acid group, p = 0.11) (Table 1). As shown in the Kaplan–Meier curve, the overall survival probability during hospital stay was statistically significant before propensity score matching among patients who used ascorbic acid; however, after propensity score matching, there was no significant difference (Fig. 1a,b). Among those who survived during their ICU stay (after PS matching), we observed that critically ill patients who received a supplemental dose of ascorbic acid as adjunctive therapy had a longer ICU LOS, and hospital LOS with a beta coefficient (95% CI) 0.47 (0.26, 0.68), p value < 0.0001, and beta coefficient (95% CI) 0.50 (0.29, 0.71), p value < 0.0001, respectively (Table 1). Among those who survived during their ICU stay (after PS matching), we observed that critically ill patients who received a supplemental dose of ascorbic acid as adjunctive therapy had a longer ICU LOS, and hospital LOS with a beta coefficient (95% CI) 0.47 (0.26, 0.68), p value < 0.0001, and beta coefficient (95% CI) 0.50 (0.29, 0.71), p value < 0.0001, respectively (Table 1).

**Table 1:** Regression analysis for the outcomes.

Outcomes	Ascorbic acid group n of outcomes/total no-of patients		p value	Odds ratio (OR) (95% CI)	p value^$^
	Control	Ascorbic Acid			
**In-hospital mortality, n (%)**^∆^					
Analysis on all eligible patients	275/558 (49.3)	50/149 (33.6)	0.0006^^^^	0.50 (0.330, 0.759)	0.001
Propensity score matched	59/142 (41.6)	46/142 (32.4)	0.11^^^^	0.77 (0.476, 1.234)	0.27
**30-day mortality**^∆^					
Analysis on all eligible patients	235/540 (43.5)	40/146 (27.4)	0.0004^^^^	0.51 (0.332, 0.794)	0.002
Propensity score matched	48/136 (35.3)	37/139 (26.6)	0.11^^^^	0.73 (0.438, 1.204)	0.21

				Beta coefficient (estimates) (95% CI)	p value^$*^
MV duration during ICU stay days, median (IQR)^&^	3.0 (1.00–11.50)	3 (0.00–12.00)	0.49^^^	0.14 (-0.24, 0.52)	0.47$^*^
ICU length of stay days, median (IQR)^&^	7.0 (4.00–12.00)	8.5 (5.00–15.00)	0.26^^^	0.47 (0.26, 0.68)	<0.0001^$*^
Hospital length of stay days, median (IQR)^&^	13.5 (10.00–23.00)	17.0 (12.00–27.00)	0.05^^^	0.73 (0.51, 0.95)	<0.0001^$*^

^∆^Denominator of the percentage is the total number of patients.

^&^Denominator is patients who survived.

^Wilcoxon rank sum test is used to calculate the p value.

^^Chi-square test is used to calculate the p value.

^$^*Propensity score adjusted Generalized linear model is used to calculate estimates and p value.

^$^Propensity score adjusted Logistic regression is used to calculate Odds ratio and p value.

### Complications during ICU stay

Complications during ICU stay were reported in (Table 2). Despite the similar use of pharmacological VTE prophylaxis (Additional file 1), we observed that patients who received ascorbic acid had a statistically significant lower rate of thrombosis/infarction compared with the non-ascorbic acid group (6.1% vs. 13%, respectively); OR (95% CI) 0.42 (0.184, 0.937), p value = 0.03. In terms of other complications during ICU stay such as respiratory failure that required MV, liver injury, and acute kidney injury were shown to be statistically insignificant between the two groups.

**Table 2:** Regression analysis for complication (s) during ICU stay.

Outcomes	Ascorbic acid group n of outcomes/total no-of patients		p value	Odds ratio (OR)(95% CI)	p value^$^
	Control	Ascorbic Acid			
**Acute kidney injury (AKI), n (%)∆**					
Analysis on all eligible patients	277/570 (48.6)	58/156 (37.2)	0.01^^^^	0.66 (0.444, 0.984)	0.04
Propensity score matched	51/146 (34.9)	56/148 (37.8)	0.60^^^^	1.34 (0.837, 2.150)	0.22
**Liver injury, n (%)∆**					
Analysis on all eligible patients	63/568 (11.1)	14/156 (9.0)	0.44^^^^	0.52 (0.277, 0.989)	0.04
Propensity score matched	9/146 (6.2)	13/148 (8.8)	0.39^^^^	1.17 (0.517, 2.653)	0.70
**Respiratory failure required MV, n (%)**^$*^					
Analysis on all eligible patients	35/156 (22.4)	27/83 (32.5)	0.12^^^^	0.97 (0.51, 1.82)	0.93
Propensity score matched	34/72 (47.2)	25/48 (52.0)	0.71^^^^	1.05 (0.51, 2.14)	0.90
**Thrombosis during ICU, n (%)∆**					
Analysis on all eligible patients	64/565 (11.3)	9/154 (5.8)	0.04^^^^	0.35 (0.167, 0.717)	0.004
Propensity score matched	19/146 (13.0)	9/147 (6.1)	0.04^^^^	0.42 (0.184, 0.937)	0.03

^∆^Denominator of the percentage is the total number of patients.

^^Chi-square is used to calculate the p value.

^$^Propensity score adjusted Logistic regression is used to calculate Odds ratio and p value.

^$*^Denominator of the percentage is non-mechanically ventilated patients with 24 h of ICU admission.

## Discussion

In this retrospective cohort study of critically ill patients with COVID-19, patients who received enteral ascorbic acid in a dose of 1000 mg daily (supplemental dose) have a similar mortality rate with patients who did not receive it.

We observed that both the in-hospital and 30-days mortality were similar in both groups after matching patients based on the severity of illness (i.e., APACHE II, SOFA score, NUTRIC scores), study center, and steroid use. More patients in the ascorbic acid group received a systematic steroid in our study than in the non-ascorbic acid group 92.9 vs. 86.5 (p value = 0.0312), respectively. This could justify ascorbic acid's survival benefit before controlling for the effect of steroid use in the overall cohort. However, the ascorbic acid group showed no mortality benefits after controlling for steroid use's potential impact.

In critically ill patients, ascorbic acid deficiency is commonly observed despite receiving proper ascorbic acid intake^16^. Furthermore, ascorbic acid deficiency is associated with multi-organ failure and increased mortality^17,18^. A bioinformatic study highlighted the potential role of ascorbic acid in sepsis. By suppressing inflammatory response and oxidative stress, which are vital pathophysiological mechanisms of sepsis, ascorbic acid may have a beneficial effect against sepsis^19^. Moreover, patients with severe COVID-19 have higher inflammatory markers and cytokine storms^20^.

Large randomized controlled studies using ascorbic acid for COVID-19 in ICU patients are lacking. One study that evaluated the use of ascorbic acid in COVID-19 patients was conducted by Jing et al. They randomized patients admitted to the ICU with COVID-19 to receive high-dose ascorbic acid (12 g) every 12 h for seven days versus placebo. This study showed no benefit to using ascorbic acid in the 28-day mortality or duration of mechanical ventilation. However, oxygenation was significantly improved in the ascorbic acid patients^12^. A recent RCT was stopped after interim analysis due to futility; high-dose zinc and vitamin C (ascorbic acid) had no impact on the course of symptoms in patients with mild COVID-19 but did not evaluate the benefits in critically ill COVID-19 patients. In addition, there was no difference in secondary endpoints, including days to symptom resolution, the severity of symptoms, hospitalizations, or deaths^21^.

The majority of our cohort have received a fixed dose of 1000 mg of ascorbic acid enterally once daily within 24 h of ICU admission with a median duration of 11 days. Even though we have utilized a lower dose than specified in the published data, our results are consistent with a recently published observational cohort study with a propensity score matching of critically ill patients who received a high dose of ascorbic acid (1.5 g of IV every 6 h) for the infection with COVID-19. This study found that an adjunctive high dose of IV ascorbic acid was not associated with mortality benefits^22^. The enteral administration of ascorbic acid might limit absorption in critically ill patients due to GI ischemia and impaired intestinal flora. However, a large meta-analysis evaluated a different route for ascorbic acid administration (IV vs. enteral) in the critically ill patients and found no significant difference in mortality between the groups but observed a tendency toward a reduction in the mortality rate with the high dose of IV ascorbic acid (RR 0.21; 95% CI 0.04–1.05; p = 0.06)^23^.

Our study shows that a low supplemental dose of enteral ascorbic acid resulted in a significant reduction in thrombosis risk during ICU stay. The underlying benefit of ascorbic acid on thrombosis could be due to its anti-inflammatory properties. Of interest is VTE prophylaxis—considered as a standard of care in our patients—and using VTE prophylaxis was similar between the two groups (p value > 0.999). Several published studies showed that the incidence of thrombosis was high in critically ill COVID-19 patients^20,24^.

Several pharmacological regimens have been proposed to positively impact the outcomes for COVID-19 patients. Out of these regimens, only dexamethasone and interleukin-6 receptor antagonists have improved the survival rate in critically ill patients^25,26^. None of the previously investigated pharmacological modalities for COVID-19 has shown a reduction in the risk of thrombosis.

Our findings showed a statistically insignificant higher rate of AKI, liver injury, and respiratory failure requiring MV in the ascorbic acid group. Even though multiple trials showed positive outcomes with ascorbic acid^8,27–30^, other trials did not improve the clinical outcomes^31,32^. In a phase I trial, 16 patients with severe sepsis received a high IV ascorbic acid dose (50–200 mg/kg/day) for four days. Ascorbic acid use showed a reduction in the sequential organ failure assessment (SOFA) score and proinflammatory biomarkers while being well-tolerated^7^. Nathens et al. used IV ascorbic acid 1 g every eight hours for 28 days in 594 critically ill surgical patients and found a significantly lower incidence of multi-organ failure, shorter mechanical ventilation duration, and ICU length of stay^15^. The lack of significant improvement in the clinical outcomes in our study could be related to the use of supplemental dose, which is a lower dose compared to previously published studies.

Ascorbic acid in non-COVID-19 patients has been studied extensively in several randomized controlled trials and observational studies with mixed results due to the lack of consistency in terms of the ascorbic acid dose, route, timing, frequency of administration in these studies, and primary outcome measures. Studies addressing ascorbic acid in COVID-19 critically ill patients are lacking. Our study provides a hypothesis-generating idea of the potential benefit of using ascorbic acid in critically ill patients with COVID-19 in reducing the risk of thrombosis. We believe that this hypothesis needs to be further investigated at a larger scale using more robust, validated modalities and study designs to eliminate the risk of bias.

Our study has several limitations in terms of the retrospective design and the heterogeneity in the comorbid conditions and disease severity that were minimized via using the propensity score. Also, baseline ascorbic acid levels were not measured before initiating the supplemental regimen, given our study's retrospective nature. Moreover, there was a dynamic change in the clinical practice of managing patients with COVID-19 as evidence continued to emerge over time. Furthermore, there was no consensus on when to start ascorbic acid, and it was mainly at the discretion of the treating team.

## Conclusion

The use of ascorbic acid was not associated with in-hospital or 30-day mortality reduction. Using low-dose ascorbic acid as adjunctive therapy in critically ill patients with COVID-19 was associated with a lower incidence of thrombosis. Further studies are warranted to confirm these findings.

### Ethics approval and consent to participate

The study was approved on November 19th, 2020, by King Abdullah International Medical Research Center (KAIMRC)-Institutional Review Board (IRB), Riyadh, Saudi Arabia (Reference No: NRC21R/286/07). All methods were performed in accordance with relevant guidelines and regulations. Participants’ confidentiality was strictly observed throughout the study by using anonymous unique serial number for each subject and restricting data only to the investigators. KAIMRC-IRB committee waived the informed consent due to its retrospective nature.

## Supplementary Information

Supplementary Information 1.

## Data Availability

The datasets used and/or analyzed during the current study are available from corresponding author on reasonable request.

## Abbreviations

ICUs: Intensive care units
COVID-19: Coronavirus disease
MV: Mechanical ventilation
LOS: Length of stay
