# Supplementary material for: Ascorbic acid as an adjunctive therapy in critically ill patients with COVID-19: a propensity score matched study
Source: Sci Rep. 2021 Sep 3;11:17648. doi: 10.1038/s41598-021-96703-y (PMC8417267; doi:10.1038/s41598-021-96703-y)
Supplement: Supplementary file 1 — Supplementary Information 1. [file 41598_2021_96703_MOESM1_ESM.docx]

**Table 1 Summary of demography and baseline characteristics**

|  | **Without Matching Using Propensity Score** | | | | **With Matching Using Propensity Score** | | | |
| --- | --- | --- | --- | --- | --- | --- | --- | --- |
|  | **Overall (739)** | **Without Ascorbic Acid (N=581)** | **Ascorbic Acid (N=158)** | **P-value** | **Overall (296)** | **Without Ascorbic Acid  (N=148)** | **Ascorbic Acid (N=148)** | **P-value** |
| **Age (Years), Mean (SD)** | 60.7 (14.81) | 60.7 (14.75) | 60.5 (15.09) | 0.8615* | 60.6 (15.15) | 60.8 (15.31) | 60.5 (15.04) | 0.7188^ |
| **Gender – Male, n (%)** | 531 (72.0) | 407 (70.1) | 124 (79.0) | 0.0271^^ | 202 (68.2) | 82 (55.4) | 120 (81.1) | <.0001^^ |
| **Weight (kg), Mean (SD)** | 81.1 (18.92) | 80.6 (18.80) | 82.8 (19.32) | 0.3258^ | 81.6 (18.62) | 80.6 (18.04) | 82.7 (19.19) | 0.8719^ |
| **Height (cm) Mean (SD)** | 163.6 (11.36) | 163.5 (11.48) | 163.9 (10.89) | 0.5164^ | 162.8 (11.57) | 161.4 (12.11) | 164.2 (10.86) | 0.0045^ |
| **Body mass index (BMI), Mean (SD)** | 30.3 (7.26) | 30.2 (7.43) | 30.6 (6.60) | 0.2781^ | 30.7 (6.87) | 31.01 (7.17) | 30.4 (6.56) | 0.3640^ |
| **GCS Baseline, Median (Q1, Q3)** | 15.0 (11.00, 15.00) | 15.0 (9.00, 15.00) | 15.0 (15.00, 15.00) | <.0001* | 15.0 (15.00, 15.00) | 15.0 (15.00, 15.00) | 15.0 (15.00, 15.00) | 0.1854^ |
| **APACHE II score, Median (Q1, Q3)** | 12.0 (8.00, 22.00) | 14.0 (8.00, 24.00) | 10.0 (7.00, 16.00) | <.0001* | 11.0 (7.00, 16.00) | 11.5 (7.00, 16.00) | 10.0(7.00, 16.00) | 0.4932^ |
| **SOFA score, Median (Q1, Q3)** | 5.0 (3.00, 8.00) | 5.0 (3.00, 8.00) | 4.0 (2.00, 6.00) | <.0001* | 4.0 (2.00, 6.00) | 4.0 (2.00, 5.50) | 4.0 (2.00, 6.00) | 0.8398^ |
| **NUTRIC Score, Median (Q1, Q3)** | 3.0 (2.00, 6.00) | 4.0 (2.00, 6.00) | 3.0 (2.00, 4.00) | 0.0002^ | 3.0 (2.00, 4.00) | 3.0 (2.00, 4.00) | 3.0 (2.00, 4.00) | 0.8173^ |
| **Systemic Corticosteroids use during ICU, n (%)** | 639 ( 87.9 ) | 495 ( 86.5 ) | 144 ( 92.9 ) | 0.0312^^ | 275 ( 92.9 ) | 138 ( 93.2 ) | 137 ( 92.6 ) | 0.8209^^ |
| **Tocilizumab Use during ICU, n (%)** | 218 ( 39.1 ) | 155 ( 38.1 ) | 63 ( 42.0 ) | 0.4008^^ | 111 ( 38.7 ) | 52 ( 35.9 ) | 59 ( 41.5 ) | 0.3226^^ |
| **Serum Creatinine (μmol/L), Median (Q1, Q3)** | 91.0 (71.00, 137.00) | 93.5 (72.00, 141.00) | 82.5 (68.00, 116.00) | 0.0143^ | 81.5 (67.00, 115.00) | 80.5 (66.00, 115.00) | 82.5 (68.00, 113.50) | 0.2513^ |
| **Urine output (UOP) (mls/kg/hour), Mean (SD)** | 0.7 (0.55) | 0.72 (0.57) | 0.7 (0.50) | 0.3800^ | 0.68 (0.52) | 0.7 (0.55) | 0.65 (0.49) | 0.3863^ |
| **Acute Kidney Injury (AKI) within 24 hours of ICU admission, n (%)** | 211 ( 28.9 ) | 178 ( 30.9 ) | 33 ( 21.3 ) | 0.0191^^ | 60 ( 20.3 ) | 29 ( 19.6 ) | 31 ( 21.1 ) | 0.7499^^ |
| **MV during ICU stay within 24 hours, n (%)** | 489 ( 66.9 ) | 396 ( 68.8 ) | 93 ( 60.0 ) | 0.0399^^ | 175 ( 59.1 ) | 86 ( 58.1 ) | 89 ( 60.1 ) | 0.7228^^ |
| **Blood sugar level (mmol/L) Baseline, Median (Q1, Q3)** | 11.7 (7.95, 16.75) | 12.0 (8.10, 17.10) | 10.8 (7.50, 15.10) | 0.0323^ | 10.9 (7.50, 15.80) | 11.0 (7.50, 16.10) | 10.70 (7.50, 15.00) | 0.5341^ |
| **Lactic acid Baseline (mmol/L), Median (Q1, Q3)** | 1.8 (1.30, 2.50) | 1.8 (1.31, 2.60) | 1.6 (1.30, 2.12) | 0.0171^ | 1.7 (1.29, 2.23) | 1.7 (1.28, 2.33) | 1.67 (1.30, 2.12) | 0.4317^ |
| **Platelets count Baseline (10^9/L), Median (Q1, Q3)** | 251.0 (191.00, 328.00) | 250.5 (189.00, 324.00) | 255.0 (204.50, 334.50) | 0.2308^ | 258.0 (201.00, 330.00) | 260.0 (201.00, 326.00) | 254.50 (202.50, 331.50) | 0.8882^ |
| **Total WBC Baseline (10^9/L), Median (Q1, Q3)** | 10.1 (7.00, 14.10) | 10.2 (7.05, 14.43) | 9.5 (6.73, 12.80) | 0.1279^ | 9.5 (6.59, 13.20) | 9.33 (6.46, 13.60) | 9.54 (6.73, 13.00) | 0.8260^ |
| **international normalized ratio (INR) , Median (Q1, Q3)** | 1.1 (1.04, 1.19) | 1.1 (1.04, 1.20) | 1.1 (1.02, 1.14) | 0.0001^ | 1.1 (1.02, 1.14) | 1.1 (1.04, 1.13) | 1.05 (1.02, 1.14) | 0.0697^ |
| **Activated partial thromboplastin time (aPTT) Baseline (Seconds), Median (Q1, Q3)** | 29.7 (26.80, 33.25) | 30.1 (27.00, 33.70) | 28.5 (25.90, 31.70) | 0.0012^ | 29.0 (26.00, 31.70) | 29.2 (26.00, 32.55) | 28.20 (25.90, 31.40) | 0.3538^ |
| **Total bilirubin (μmol/L), Median (Q1, Q3)** | 10.0 (7.00, 14.60) | 10.0 (7.00, 15.00) | 10.0 (7.00, 13.70) | 0.5515^ | 10.3 (7.25, 14.60) | 10.3 (7.80, 15.40) | 10.10 (7.20, 14.00) | 0.5113^ |
| **Alanine aminotransferase (ALT) Baseline (U\L), Median (Q1, Q3)** | 38.0 (25.00, 66.00) | 38.0 (25.00, 64.00) | 41.0 (29.00, 69.00) | 0.2910^ | 38.0 (25.00, 66.00) | 35.0 (24.00, 60.50) | 41.00 (29.00, 69.00) | 0.0749^ |
| **Aspartate aminotransferase (AST) Baseline (U\L), Median (Q1, Q3)** | 55.0 (35.00, 80.00) | 54.0 (35.00, 81.00) | 57.0 (35.00, 80.00) | 0.6678^ | 53.0 (34.00, 76.00) | 50.0 (33.00, 75.00) | 57.00 (35.00, 79.00) | 0.1735^ |
| **Albumin Baseline (gm/L), Median (Q1, Q3)** | 33.0 (30.00, 36.30) | 34.0 (29.00, 37.00) | 33.0 (30.00, 36.00) | 0.2692^ | 33.0 (31.00, 36.00) | 34.0 (31.00, 36.00) | 33.00 (30.00, 36.00) | 0.2628^ |
| **Blood urea nitrogen (BUN) Baseline (mmol/L), Median (Q1, Q3)** | 7.2 (4.90, 12.30) | 7.3 (5.00, 13.00) | 6.7 (4.30, 10.40) | 0.0197^ | 6.7 (4.40, 10.20) | 6.7 (4.65, 9.95) | 6.7 (4.30, 10.20) | 0.9695^ |
| **Creatine phosphokinase (CPK) (U/l), Median (Q1, Q3)** | 200.5 (78.00, 514.00) | 194.0 (77.50, 522.50) | 228.5 (85.00, 477.00) | 0.5742^ | 188.5 (77.50, 416.50) | 169.0 (62.00, 313.00) | 229.0 (90.00, 477.00) | 0.0689^ |
| **C-reactive protein (CRP) (mg/l), Median (Q1, Q3)** | 156.0 (86.00, 224.00) | 160.0 (92.00, 234.00) | 136.0 (77.00, 187.00) | 0.0490^ | 136.0 (77.00, 205.00) | 138.0 (74.00, 241.00) | 136.0 (78.00, 185.00) | 0.6667^ |
| **Procalcitonin (ng/ml), Median (Q1, Q3)** | 0.4 (0.15, 1.38) | 0.4 (0.16, 1.51) | 0.3 (0.12, 0.69) | 0.0069^ | 0.3 (0.13, 0.73) | 0.3 (0.14, 0.64) | 0.3 (0.12, 0.76) | 0.8077^ |
| **Ferritin (ug/l), Median (Q1, Q3)** | 856.9 (414.20, 2026.40) | 875.2 (425.30, 2061.10) | 833.9 (392.90, 1847.80) | 0.5015^ | 785.3 (384.30, 1858.10) | 680.1 (298.70, 1668.50) | 895.9 (448.60, 1908.40) | 0.0533^ |
| **Fibrinogen Level (gm/l), Median (Q1, Q3)** | 6.7 (4.93, 332.00) | 7.1 (5.01, 433.00) | 5.6 (4.50, 6.86) | 0.0001^ | 5.5 (3.99, 6.76) | 5.5 (3.89, 6.78) | 5.6 (4.36, 6.73) | 0.5850^ |
| **Bicarbonate (CO2) Baseline (mmol/L), Median (Q1, Q3)** | 21.0 (19.00, 24.00) | 21.0 (18.30, 24.00) | 21.0 (19.00, 24.00) | 0.8220^ | 21.0 (19.00, 24.00) | 22.0 (19.00, 24.00) | 21.0 (19.00, 24.00) | 0.6054^ |
| **Chloride (CL) Baseline (mmol/L), Median (Q1, Q3)** | 102.0 (99.00, 106.00) | 102.0 (99.00, 106.00) | 103.0 (99.00, 106.00) | 0.8477^ | 102.0 (99.00, 106.00) | 102.0 (99.00, 106.00) | 103.0 (99.00, 106.00) | 0.4226^ |
| **Potassium (K) Baseline (mmol/L), Median (Q1, Q3)** | 4.2 (3.75, 4.75) | 4.2 (3.72, 4.90) | 4.1 (3.80, 4.50) | 0.0077^ | 4.1 (3.70, 4.60) | 4.2 (3.70, 4.70) | 4.0 (3.80, 4.50) | 0.0926^ |
| **Sodium baseline (mmol/L), Median (Q1, Q3)** | 137.0 (134.75, 140.75) | 137.0 (134.00, 141.00) | 137.50 (135.00, 140.00) | 0.6743^ | 137.0 (135.00, 140.00) | 137.0 (135.00, 139.75) | 137.5 (135.00, 140.00) | 0.4412^ |
| **Hematocrit (Hct) (L\L), Median (Q1, Q3)** | 0.4 (0.35, 0.47) | 0.4 (0.35, 0.50) | 0.4 (0.36, 0.44) | 0.1538^ | 0.39 (0.35, 0.43) | 0.38 (0.33, 0.41) | 0.40 (0.36, 0.44) | 0.0021^ |
| **PaO2/FiO2 ratio within 24 hours of admission, Median (Q1, Q3)** | 75.0 (50.75, 115.00) | 73.0 (47.00, 118.47) | 79.0 (57.89, 107.00) | 0.1732^ | 81.3 (58.89, 119.88) | 85.1 (61.18, 137.50) | 79.4 (57.89, 108.00) | 0.2803^ |
| **Heart rate (HR) Baseline (BPM), Median (Q1, Q3)** | 103.0 (91.00, 115.00) | 104.0 (93.00, 116.00) | 102.0 (89.00, 114.00) | 0.1128^ | 103.0 (91.00, 116.00) | 102.5 (92.00, 118.00) | 103.0 (90.00, 115.00) | 0.2520^ |
| **Lowest MAP Baseline (mmhg), Median (Q1, Q3)** | 72.0 (63.00, 83.00) | 72.00 (63.00, 83.00) | 73.0 (65.00, 84.00) | 0.3448^ | 72.0 (64.00, 82.00) | 71.0 (63.00, 81.00) | 73.0 (65.00, 84.00) | 0.1079^ |
| **Respiratory rate (RR) Baseline (Breath per minute), Median (Q1, Q3)** | 29.0 (24.00, 35.00) | 28.0 (24.00, 34.00) | 30.0 (25.00, 35.00) | 0.1702^ | 30.0 (24.00, 35.00) | 29.5 (24.00, 35.00) | 30.0 (25.00, 35.00) | 0.8875^ |
| **Maximum temperature Baseline (C°), Median (Q1, Q3)** | 37.4 (37.00, 38.10) | 37.5 (37.00, 38.25) | 37.2 (37.00, 38.00) | 0.0204^ | 37.40 (37.00, 38.00) | 37.40 (37.10, 38.10) | 37.20 (37.00, 38.00) | 0.0980^ |
| **Pharmacological DVT prophylaxis, n (%)** | 738 (99.8) | 489 (84.1) | 148 (94.3) | 0.001 | 296 (100) | 139 (93.9) | 139 (93.9) | >0.999^^ |
| **Coexisting illness** |  |  |  |  |  |  |  |  |
| **Dyslipidemia (DLP)** | 168 (23.2) | 122 (21.4) | 46 (29.5) | 0.0349^^ | 85 (29.0) | 43 (29.7) | 42 (28.4) | 0.8097^^ |
| **Diabetes mellitus (DM)** | 442 (61.0) | 348 (61.2) | 94 (60.3) | 0.8376^^ | 173 (59.0) | 83 (57.2) | 90 (60.8) | 0.5345^^ |
| **Hypertension (HTN)** | 412 (56.8) | 324 (56.9) | 88 (56.4) | 0.9054^^ | 165 (56.3) | 83 (57.2) | 82 (55.4) | 0.7514^^ |
| **Acute Coronary Syndrome (ACS), n (%)** | 12 (1.7) | 10 (1.8) | 2 (1.3) | >0.9999** | 4 (1.4) | 2 (1.4) | 2 (1.4) | >0.9999** |
| **Asthma, n (%)** | 62 (8.6) | 45 (7.9) | 17 (10.9) | 0.2421^^ | 30 (10.3) | 13 (9.1) | 17 (11.5) | 0.5017^^ |
| **Atrial fibrillation (AFib.), n (%)** | 20 (2.8) | 18 (3.2) | 2 (1.3) | 0.2752** | 7 (2.4) | 5 (3.5) | 2 (1.4) | 0.2773** |
| **Chronic obstructive pulmonary disease (COPD)** | 15 (2.1) | 12 (2.1) | 3 (1.9) | >0.9999** | 8 (2.8) | 5 (3.5) | 3 (2.0) | 0.4965** |
| **Cancer** | 26 (3.6) | 21 (3.7) | 5 (3.2) | 0.7762^^ | 8 (2.8) | 4 (2.8) | 4 (2.7) | >0.9999** |
| **Chronic kidney disease (CKD)- (Non-Dialysis)** | 53 (7.3) | 43 (7.6) | 10 (6.5) | 0.6902** | 20 (6.9) | 11 (7.7) | 9 (6.1) | 0.9443** |
| **Chronic kidney disease (CKD)- (On Dialysis)** | 25 (3.5) | 22 (3.9) | 3 (1.9) | 0.6902** | 6 (2.1) | 3 (2.1) | 3 (2.0) | 0.9443** |
| **Coronary artery bypass grafting (CABG)** | 21 (2.9) | 18 (3.2) | 3 (1.9) | 0.5913** | 6 (2.1) | 3 (2.1) | 3 (2.0) | >0.9999** |
| **Heart failure (HF)** | 59 (8.0) | 52 (8.9) | 7 (4.4) | 0.0844** | 17 (5.7) | 11 (7.4) | 6 (4.1) | 0.0816** |
| **Hypothyroidism** | 44 (6.1) | 33 (5.8) | 11 (7.1) | 0.5525^^ | 20 (6.9) | 10 (6.9) | 10 (6.8) | 0.9619^^ |
| **Ischemic heart disease (IHD)** | 63 (8.5) | 50 (8.6) | 13 (8.2) | 0.4652** | 23 (7.8) | 11 (7.4) | 12 (8.1) | >0.9999** |
| **Liver disease (any type)** | 14 (2.0) | 9 (1.5) | 5 (3.2) | 0.4956** | 8 (2.7) | 3 (2.0) | 5 (3.4) | 0.4475** |
| **Venous thromboembolism (VTE) (PE_DVT)** | 9 (1.2) | 7 (1.2) | 2 (1.3) | >0.9999** | 3 (1.0) | 1 (0.7) | 2 (1.4) | >0.9999** |
| ^ Wilcoxon rank sum test is used to calculate the P-value.  ^^ Chi square/ ** Fisher’s Exact teat is used to calculate P-value. | | | | | | | | |
